# From COVID-19 to Influenza—Real-Life Clinical Practice in a Pediatric Hospital

**DOI:** 10.3390/diagnostics12051208

**Published:** 2022-05-12

**Authors:** Victor Daniel Miron, Gabriela Bar, Claudiu Filimon, Mihai Craiu

**Affiliations:** 1 Carol Davila University of Medicine and Pharmacy, 050474 Bucharest, Romania; claudiu.filimon@stud.umfcd.ro (C.F.); mihai.craiu@umfcd.ro (M.C.); 2 National Institute for Mother and Child Health “Alessandrescu-Rusescu”, 020395 Bucharest, Romania; bargabriela@yahoo.com

**Keywords:** children, COVID-19, influenza, omicron, SARS-CoV-2

## Abstract

The COVID-19 pandemic, through the restrictions and the non-pharmaceutical interventions implemented, has importantly impacted the circulation and epidemiology of respiratory viruses. Specifically, the 2020/21 season was entirely dominated by SARS-CoV-2, while influenza activity reached an all-time low, despite initial warnings that a double concurrent epidemic could be possible. The current season, 2021/22, started with the shift of circulating SARS-CoV-2 variants from delta to omicron, which then rapidly spread globally, as most countries, including Romania, removed all restrictions and compulsory non-pharmaceutical interventions. In this report we present the clinical reality observed in March 2022 in a tertiary paediatric hospital in Bucharest, Romania, where we observed a sudden surge in influenza cases, after two consecutive years (March 2020 to March 2022) when influenza had stopped circulating in our country. Thus, in March 2022 the positivity rate of rapid influenza antigen tests unexpectedly increased to 33.5%, paralleled by a decrease to 7.5% in the positivity rate of rapid SARS-CoV-2 antigen tests. This significant increase in the influenza attack rate was observed from the first week (14.9% positivity rate), through the fourth week of March (42.1% positivity rate, *p* < 0.001), while the COVID-19 attack rate displayed a significant decreasing trend (from 11.2% to 4.8%, *p* < 0.001). These data serve as a warning about relaxing restrictions in a precipitous approach with minimised vigilance. The evolution of these observations needs to be followed very carefully in all countries, particularly in settings where epidemiological interactions and non-pharmaceutical interventions have so far led to the extensive circulation of only one of these viruses, and we should now be prepared to perform a correct differential diagnosis between influenza and COVID-19, in order to ensure the best quality of care and personalized management of each case of respiratory infection. The results of active influenza surveillance studies for the whole 2021/22 season are awaited, in order to quantify the joint influenza—COVID-19 burden among children.

## 1. Introduction

The year 2020 has started with a sense of uncertainty as severe acute respiratory syndrome coronavirus 2 (SARS-CoV-2) infections spread with increasing alarm. Measures taken immediately after the identification of a new viral pathogen failed to isolate the outbreak in China, and it quickly spread globally so that in March 2020 the World Health Organization (WHO) declared a pandemic [1]. Subsequently, drastic containment measures to limit SARS-CoV-2 infections were adopted by almost all governments worldwide, including lockdowns, mandatory mask wearing and social distancing.

As the pandemic and the number of COVID-19 cases have progressed, there have been important changes in the circulation of other seasonal viruses. The lockdown and its associated public health measures have to a certain extent played an important role in this transformation. A sudden disappearance of influenza and RSV cases has been particularly evident in the paediatric population. Overall, there was a significant decrease in the number of cases of acute viral infections among children, also quantified by the reduced number of paediatric emergencies in the early months of the pandemic [2,3,4,5].

Understanding the mechanisms underlying the observed changes in the circulation of seasonal viruses in children and beyond is of great interest. That is why throughout the pandemic there has been an active surveillance of the other seasonal viruses in addition to SARS-CoV-2, correlated with the dynamics of existing restrictions and non-pharmacological measures.

While certain respiratory pathogens such as rhinovirus continued to circulate throughout the pandemic, the epidemiology of other viruses, particularly influenza viruses, was specifically impacted in the first two years of the pandemic [6,7]. As a result, the 2020/21 cold season in Romania and much of Europe was entirely dominated by SARS-CoV-2, while influenza activity reached an all-time low, despite initial warnings that a double concurrent epidemic could be possible.

In this brief communication we present clinical real-world data from March 2022 in a tertiary paediatric hospital in Bucharest, Romania, where we observed a sudden surge in influenza cases, after two consecutive years (March 2020 to March 2022) when influenza had stopped circulating in our country. We also highlight the most important aspects of viral circulation during the pandemic setting among children and discuss further directions on surveillance of viral infections in the paediatric population.

## 2. Clinical Practice Observations

The National Institute for Mother and Child Health (NIMCH) “Alessandrescu-Rusescu” is a tertiary paediatric hospital in the capital of Romania, Bucharest. This hospital mainly serves the paediatric population in the northern area of the city, together with the urban area of Voluntari-Pipera. In total, about 40,000 children present annually to the NIMCH Emergency Department (ED). 

The first four weeks of March 2022 were very busy for the medical team working in the ED, with a significant increase in the number of presentations (*n* = 4941, Table 1) compared to previous years (3154 in 2019, and 1923 in 2020) [5]. Our hospital is able to perform a range of investigations for children assessed in the ED, including rapid antigen detection testing (RADT) for influenza, SARS-CoV-2, respiratory syncytial virus (RSV), adenovirus or group A *Streptococcus*. Given the epidemiological situation most children with respiratory symptoms (cough, nasal obstruction, dyspnoea) and fever are evaluated by RADT from nasopharyngeal swab for influenza and SARS-CoV-2. In our hospital consistent testing for influenza, during the current season, began in January 2022 and was carried out in parallel with testing for SARS-CoV-2. 

The wave of omicron has led to an increase in COVID-19 cases in our country starting in late December 2021. Children were more commonly affected in this particular wave compared to previous ones [8,9]. This trend has also been observed in our hospital where the positivity rates of SARS-CoV-2 tests performed among children evaluated in the ED had also reached 33.7% in January 2022. In February the trend of COVID-19 cases started to decrease both globally [10] and in our country [8], which was also apparent among children evaluated in our ED. However, we continued to actively monitor and test all suspected cases for COVID-19 and influenza, so that on 18 February 2022 we identified the first child who tested positive for influenza A. In the following days, we observed sporadic cases of influenza (15 cases in 11 days) and a continued decrease in cases positive for SARS-CoV-2. 

**Figure 1:**
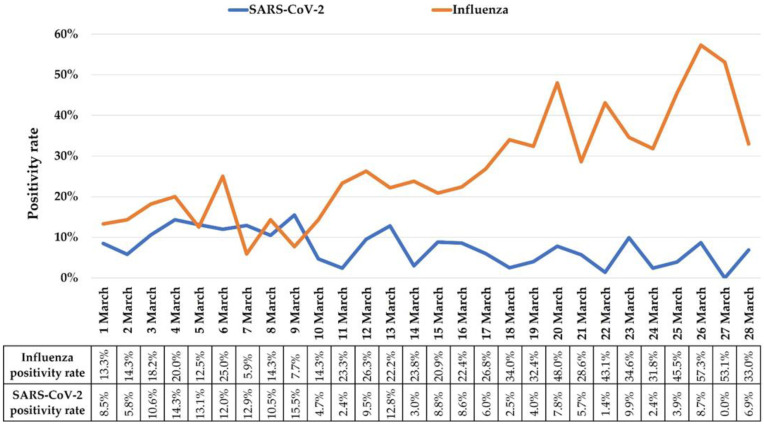
Daily distribution of influenza and SARS-CoV-2 positivity rates.

We performed an analysis of all consecutive children (under 18 years) assessed in the ED between 1–28 March 2022. A total of 1253 influenza RADT (Rapid flu test BioTracer™, South Korea; sensitivity: 95.1% for influenza A and 95.9% for influenza B; specificity 99.9%, [11]) were performed and 33.5% (*n* = 420) were positive (394 influenza A cases, 26 influenza B cases). In contrast, during the same period, a total of 1979 SARS-CoV-2 RADT (Clungene COVID-19 Antigen Rapid Test, Hangzhou Clongene Biotech, Hangzhou, China; sensitivity: 96.7%; specificity 100%, [12]) were performed, 7.5% (*n* = 149) were positive (*p* < 0.001, χ^2^ = 357.3, OR = 6.2, 95%CI: 5.0–7.6). Table 1 details the total number of children evaluated in the ED, as well as the number of children tested for COVID-19 and influenza on each day in March. In addition, in Figure 1 we have highlighted in comparison the positivity rates for influenza and COVID-19 for each day analysed. A significant increase in influenza cases was observed from the first week of March (14.9% positivity rate, *n* = 13/87), to the fourth week (42.1% positivity rate, *n* = 247/587, *p* < 0.001, χ^2^ = 23.6, OR = 4.1, 95%CI: 2.2–7.6), while COVID-19 cases had a significant decreasing trend (from 11.2% positivity rate in the first week, *n* = 52/466, to 4.8% positivity rate in the fourth week of March, *n* = 25/526, *p* < 0.001, χ^2^ = 14.2, OR = 2.5, 95%CI: 1.5–4.1). The median age of patients testing positive for influenza was 5 years (IQR: 3, 8), with a slight male predominance (59.5%, *n* = 250). Only 10 children with influenza (2.4%) required hospitalization. In all non-hospitalized cases, parents were provided with a written action-plan for their children, a list of warning signs, and all parents were advised to maintain protective measures and isolation in order to limit transmission of the influenza virus.

The high number of presentations to the ED was paralleled by the increasing number of influenza cases. We were consistent in following up cases of influenza and COVID-19 among children in order to ensure proper case management and take epidemiological action accordingly.

## 3. Circulation of Influenza Viruses during the Pandemic and Future Prospects

Less than 3 months elapsed between the appearance of the first cases of COVID-19 and proclaiming pandemic status by WHO, thus highlighting the threat of impending active community transmission of SARS-CoV-2 in many countries worldwide [1]. This led, as shown above, to a rapid enforcement of lockdown and strict epidemiological measures that also impacted the circulation of other respiratory viruses. Thus, immediately after the restrictions were instituted, in March-April 2020, reductions to zero of influenza cases were reported in many countries from different continents: the USA [13], Canada [14], Japan [15], China [16], Italy [16] and Romania [17].

The cold season 2020/21 raised concerns about a possible double SARS-CoV-2—influenza epidemic, so that the general recommendations from WHO and the local authorities were to strictly adhere to non-pharmacological interventions designated to prevent the spread of respiratory viruses. In addition, influenza vaccination campaigns were deployed especially for at-risk groups [18]. Overall, in the 2020/21 season there was an increased interest regarding influenza vaccine [19] and its uptake increased among various population groups [20]. As a result of the measures implemented, while SARS-CoV-2 was still circulating at high intensity, during that period “influenza activity was lower than during any previous influenza season” [7]. In the USA out of over one million samples tested for influenza 0.2% were positive [7], and in Europe the number of positive cases decreased by 99.5% compared to previous seasons [21]. Similar data, with no outbreaks of seasonal influenza, were reported in China [22], Japan [23] and Brazil [24]. 

Meanwhile, the pandemic has also had a significant impact on the circulation of other respiratory viruses. From the onset of the pandemic until spring 2021, low circulation rates of RSV, common coronaviruses, parainfluenza viruses, metapneumovirus or adenovirus were recorded [7]. Rhinovirus circulation appears to have been less affected by pandemic and non-pharmacological interventions [7,25] and continued to cause respiratory infections among children as well as adults.

Another significant impact of the COVID-19 pandemic on respiratory viruses was the change in seasonal circulation patterns. This was most evident for RSV, which unexpectedly prompted an epidemic of cases among young children worldwide in the 2021 warm season. The first evidence of the RSV viral activity in 2021 came from the Southern Hemisphere, where in Australia, after restrictions were eased, a wave of RSV illnesses emerged [26]. As of April 2021, numerous outbreaks of RSV infections have been reported in many Northern hemisphere countries [27,28,29].

The 2021/22 influenza season started with the change of circulating SARS-CoV-2 variants from delta to omicron. Omicron spread globally very fast due to its high transmissibility rate, but fortunately the severity of cases was lower than of previous variants. In Romania, from the first community-transmitted case reported in December 2021 [30], omicron reached tens of thousands of cases in January, and then followed a downward trend [8] as globally [10]. In this context, although general interest in influenza has diminished, the influenza infrastructure has remained active in surveillance institutions. In addition, in most countries, as well as in Romania, most restrictions were eased and wearing masks in public places became optional. Thus, a rapid “relaxation” without a contingency plan was unfortunately followed by a new wave of respiratory illnesses that is now affecting the paediatric population in particular. As we reported in this communication, EDs have been recently overwhelmed by large numbers of children, and the influenza has quickly made its presence felt. SARS-CoV-2 has however not disappeared either, in spite of the significant decline of cases reported in current wave, but it has remained present at a low baseline level (4.7% attack rate in the last week of March 2022). This is why special attention, as well as a contingency plan, are needed in the near future.

The children population is of particular relevance when studying influenza and COVID-19, for several reasons. On the one hand, the World Health Organization lists children aged 6 to 59 months among the risk groups for influenza, highlighting the importance of prioritizing them for yearly influenza vaccination, as this age group carries a high burden of influenza-associated hospitalizations. At the same time, epidemiological studies show that each season among patients hospitalized for influenza, children represent a significant percentage [17,31,32]. On the other hand, while the initial waves of COVID-19 concentrated most of the morbidity and mortality among elderly patients, children were not spared, with reports from Latin America indicating that 47% of confirmed paediatric cases required hospitalization, and among these 16% required intensive care, particularly infants, or children of all ages with underlying diseases [33]. With the emergence of the omicron variant in late 2021, the overall number of cases of COVID-19 in children increased importantly, and so did the paediatric rate of intensive care admission, reported at 19% for COVID-19 in children 5 to 11 years of age during the omicron wave in the USA [34]. 

We have now witnessed a renewed influenza circulation among children in settings where influenza viruses have not circulated for the largest part of the past two years. Since these two infections can have similar initial presenting complaints, this emphasizes the importance of being prepared to perform a correct differential diagnosis between influenza and COVID-19 in paediatric healthcare and beyond, to ensure correct monitoring and prompt access to appropriate antiviral treatment. Furthermore, we should keep in mind that influenza has long been considered a vaccine-preventable illness, and that everyone aged 6 months and above can benefit from vaccination, and this is also increasingly true for COVID-19, with vaccination now being available for children aged 5 years and older, and clinical trials are ongoing for younger ages. 

As shown in a recent report, influenza co-infection in COVID-19 patients is associated with a higher rate of ICU admission and mortality [35]. Under these circumstances, despite of physical and mental exhaustion induced by restrictions taken since the onset of the pandemic, the general population has to understand that the pandemic is not over, and the influenza viral circulation is far from being over and uncertainty persists. Non-pharmacological measures have proven their effectiveness in limiting the transmission of respiratory infections and are the easiest measure to prevent future outbreaks.

Integrated testing and surveillance of influenza viruses and SARS-CoV-2 is a very clear need in the current pandemic. Detection of other respiratory viruses is also essential for proper patient management. We believe that this is currently achievable given the possibility of RADT and molecular detection by multiplex RT-PCR by most hospitals from our country. We also believe that where these tools are not available, there is a great need for such cost-efficient instruments. Spatial and temporal mapping of respiratory illnesses especially among children in the next future will help evidence-based decision making and implementation of measures to limit possible outbreaks of respiratory diseases. The circulation of influenza in the off-season is not to be neglected, given the experience of RSV in 2021 [36]. Therefore, although the return of influenza is certain, the further evolution of the behaviour of influenza viruses is uncertain. There is a need to continue vaccination against COVID-19 and influenza, including children, as the burden on health systems of these infections is large, both directly and indirectly.

## 4. Conclusions

In the clinical setting of an ED of a tertiary paediatric hospital, we observed an increased number of influenza cases starting in March 2022, in parallel with a slightly decreasing number of COVID-19 cases. These data serve as a warning about relaxing restrictions in a precipitous approach with minimised vigilance. The evolution of these observations needs to be followed very carefully in all countries, particularly in settings where epidemiological interactions and non-pharmaceutical interventions have so far led to the extensive circulation of only one of these viruses, and we should now be prepared to perform a correct differential diagnosis between influenza and COVID-19, in order to ensure the best quality of care and personalized management. The results of active influenza surveillance studies for the whole 2021/22 season are awaited, in order to quantify the joint influenza—COVID-19 burden among children.

## Figures and Tables

**Table 1:** Daily number of cases evaluated in the Emergency Department and tested for influenza and COVID-19.

Date		Total Number of Cases	Children Tested for SARS-Cov-2	COVID-19 Positive	Children Tested for Influenza	Influenza Positive	Influenza A	Influenza B
		*n*	*n* (%) *	*n* (%) **	*n* (%) *	*n* (%) ***	*n*	*n*
March	1	126	47 (37.3)	4 (8.5)	15 (11.9)	2 (13.3)	2	0
	2	137	69 (50.4)	4 (5.8)	14 (10.2)	2 (14.3)	2	0
	3	140	66 (47.1)	7 (10.6)	11 (7.9)	2 (18.2)	2	0
	4	152	70 (46.1)	10 (14.3)	10 (6.6)	2 (20.0)	2	0
	5	130	61 (46.9)	8 (13.1)	8 (6.2)	1 (12.5)	1	0
	6	139	83 (59.7)	10 (12.0)	12 (8.6)	3 (25.0)	3	0
	7	166	70 (42.2)	9 (12.9)	17 (10.2)	1 (5.9)	1	0
	8	131	76 (58.0)	8 (10.5)	14 (10.7)	2 (14.3)	2	0
	9	171	58 (33.9)	9 (15.5)	13 (7.6)	1 (7.7)	1	0
	10	150	43 (28.7)	2 (4.7)	14 (9.3)	2 (14.3)	2	0
	11	181	85 (47.0)	2 (2.4)	30 (16.6)	7 (23.3)	6	1
	12	177	63 (35.6)	6 (9.5)	38 (21.5)	10 (26.3)	9	1
	13	167	78 (46.7)	10 (12.8)	36 (21.6)	8 (22.2)	8	0
	14	210	67 (31.9)	2 (3.0)	42 (20.0)	10 (23.8)	9	1
	15	194	102 (52.6)	9 (8.8)	43 (22.2)	9 (20.9)	7	2
	16	171	81 (47.4)	7 (8.6)	49 (28.7)	11 (22.4)	11	0
	17	192	84 (43.8)	5 (6.0)	56 (29.2)	15 (26.8)	14	1
	18	198	79 (39.9)	2 (2.5)	53 (26.8)	18 (34.0)	18	0
	19	176	50 (28.4)	2 (4.0)	71 (40.3)	23 (32.4)	23	0
	20	198	51 (25.8)	4 (7.8)	50 (25.3)	24 (48.0)	23	1
	21	228	70 (30.7)	4 (5.7)	70 (30.7)	20 (28.6)	20	0
	22	199	72 (36.2)	1 (1.4)	72 (36.2)	31 (43.1)	30	1
	23	180	81 (45.0)	8 (9.9)	81 (45.0)	28 (34.6)	28	0
	24	213	82 (38.5)	2 (2.4)	88 (41.3)	28 (31.8)	28	0
	25	228	77 (33.8)	3 (3.9)	77 (33.8)	35 (45.5)	35	0
	26	189	46 (24.3)	4 (8.7)	96 (50.8)	55 (57.3)	54	1
	27	185	66 (35.7)	0 (0.0)	64 (34.6)	34 (53.1)	32	2
	28	213	102 (47.9)	7 (6.9)	109 (51.2)	36 (33.0)	35	1
Total		4941	1979 (40.1)	149 (7.5)	1253 (25.4)	420 (33.5)	408	12

*—Percentage calculated from the total number of patients, **—Percentage calculated from the number of patients tested for SARS-CoV-2, ***—Percentage calculated from the number of patients tested for influenza.

## Data Availability

The datasets generated and analysed during the current study are available from the corresponding author upon reasonable request.
